# Supplementary figures and images for: Solid Organ Transplantation Is Associated with an Increased Rate of Mismatch Repair Deficiency and PIK3CA Mutations in Colorectal Cancer
Source: Curr Oncol. 2022 Dec 21;30(1):75–84. doi: 10.3390/curroncol30010006 (PMC9858144; doi:10.3390/curroncol30010006)

Supplemental Figure S1

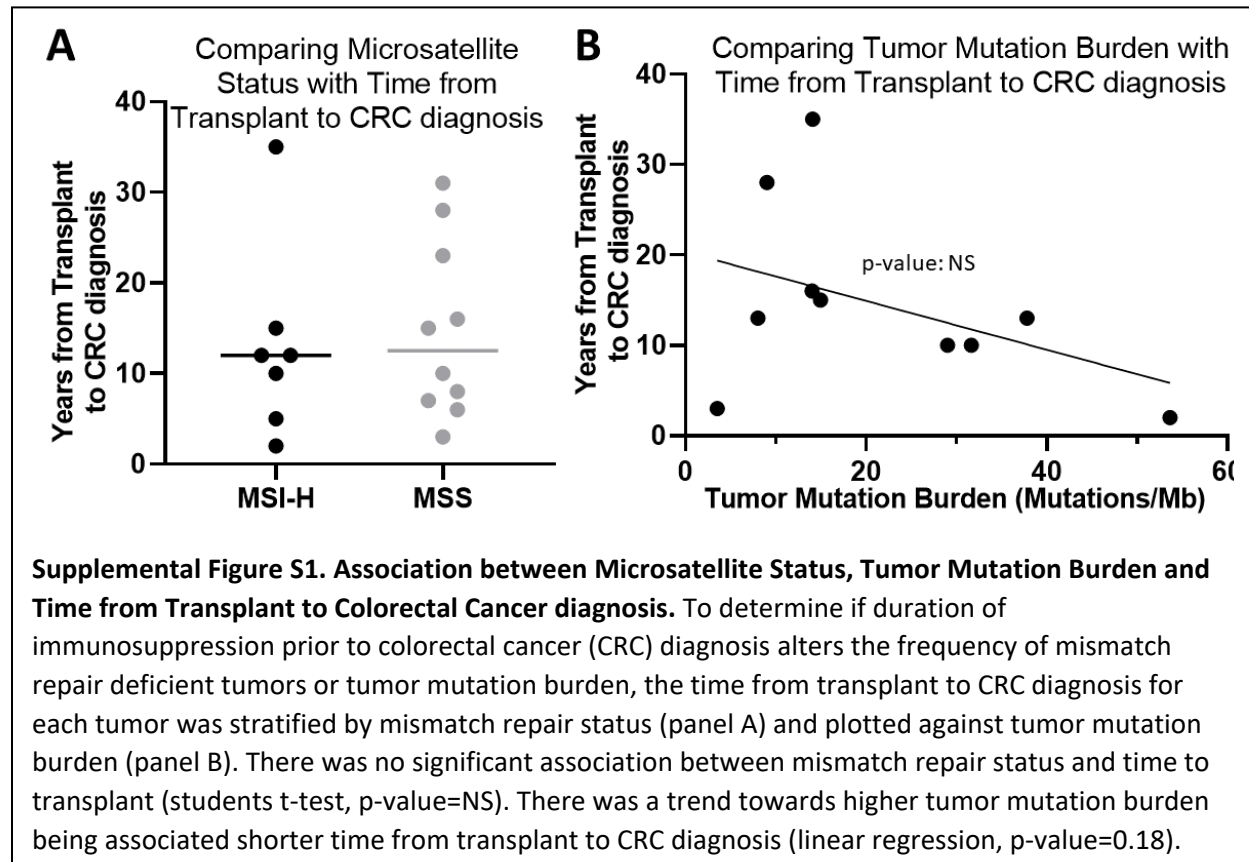

Supplement: Supplementary file 1 [file curroncol-30-00006-s001.zip › curroncol-2018102-supplementary.pdf]
